# Effects of Surface Functionalization of Lignin on Synthesis and Properties of Rigid Bio-Based Polyurethanes Foams

**DOI:** 10.3390/polym10070706

**Published:** 2018-06-26

**Authors:** Xuefeng Zhang, Dragica Jeremic, Yunsang Kim, Jason Street, Rubin Shmulsky

**Affiliations:** Department of Sustainable Bioproducts, Mississippi State University, Mississippi State, MS 39762, USA; dragica.jn@gmail.com (D.J.); ysk13@msstate.edu (Y.K.); jason.street@msstate.edu (J.S.)

**Keywords:** lignin, surface functionalization, rigid polyurethane foam, compressive strength and modulus, thermal insulation, building materials

## Abstract

We report the preparation of lignin-based rigid polyurethane (RPU) foams from surface functionalized kraft lignin via a simple and environmentally benign process. Lignin was functionalized with polyisocyanate at 80 °C for 1 h, the resulting lignin-polyisocyanate prepolymer was confirmed by increased viscosity and Fourier-transform infrared spectroscopy (FTIR). The RPU foams containing up to 30% surface functionalized lignin as a substitute for petroleum-based polyols exhibited comparable thermal and mechanical properties to conventional RPU foams. The lignin-based RPU foams prepared from surface functionalization outperformed RPU foams without the surface functionalization, showing up to 47% and 45% higher specific compressive strength and modulus, respectively, with a 40% lignin substitution ratio. Thermal insulation and temperature-stability of the two types of the foams were comparable. The results indicate that the surface functionalization of lignin increases reactivity and homogeneity of the lignin as a building block in RPU foams. The life cycle assessment for the lignin-based RPU foams shows that the surface functionalization process would have overall lesser environmental impacts when compared with the traditional manufacturing of RPU foams with synthetic polyols. These findings suggest the potential use of surface functionalized lignin as a sustainable core material replacement for synthetic polyols in building materials.

## 1. Introduction

Rigid polyurethane (RPU) foams are one of the most commonly used polymeric materials in building applications such as wall panels, flooring, and structural insulated panels (SIPs) because of their low density, high dimensional stability, good adhesion, and excellent thermal insulation and mechanical properties [1]. Despite their superior properties, the use of petroleum-based polyols for the production of the RPU foams can be problematic due to the depletion of fossil fuel resources and negative environmental impacts of the industrial production of synthetic polyols [2]. Therefore, the development of renewable bio-based polyols derived from natural resources has garnered a strong global interest both in academia and industry.

Lignin is the most abundant aromatic biopolymer on Earth and is available in large quantities from wood-pulping and bio-ethanol industries [3,4,5]. Extensive efforts have been made to convert lignin to polyurethane compounds because lignin contains a large amount of aliphatic and aromatic hydroxyl groups [6,7,8]. The use of lignin as a polyol substitute in the preparation of RPU foams commonly follows two general approaches. In the first approach, lignin undergoes chemical modification processes, i.e., oxypropylation and liquefaction, to increase the number of hydroxyl groups with enhanced accessibility for reactions with polyisocyanates [9,10,11,12,13,14,15]. Although this approach increases the reactivity of lignin, it is difficult to prepare RPUs containing relatively high quantities of lignin [16]. In addition, the chemical modification processes increase the production cost due to the extensive use of energy and larger space requirements for accommodation of equipment [17], such as high pressure or microwave reactors [1,18], thereby making this approach less attractive to the polyurethane industries [17]. Increased environmental footprint from the chemical modification of lignin due to the use of strong acid or toxic substances is an additional concern. Alternatively, RPU foams can also be prepared by a relatively simple approach, in which unmodified lignin is mixed with petroleum-based polyols and directly combined with polyisocyanates. Various unmodified types of lignin i.e., lignosulfonates, kraft, hydrolysis, and organosolv lignin, have been used to replace up to 40 wt. % of petroleum-based polyols in RPU foams [7,19,20,21,22]. Although this simple and fast approach is appealing because of its potential to reduce the process cost, the use of unmodified lignin as a polyol substitute often negatively impacts the mechanical properties of the RPU foams [7,20,23]. The reduced mechanical properties of the lignin-containing RPU foams are generally attributed to low reactivity of lignin with polyisocyanates and to poor dispersion of lignin in the cellular structure of the foams [7,20].

**Scheme 1:**
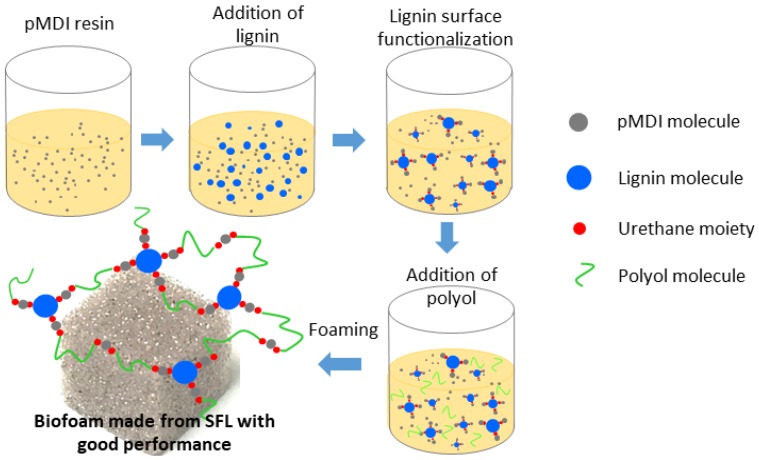
Illustration of the process and formation mechanism of surface functionalized lignin-based rigid polyurethane (SFL-RPU) foam.

In the present study, in order to overcome cost-associated issues related to lignin oxypropylation and liquefaction as well as the poor mechanical properties of unmodified lignin-based RPU foams, methyl diphenyl diisocyanate (MDI)-based polyisocyanate (pMDI) was used to functionalize kraft lignin. The highly reactive isocyanate group in pMDI can interact with lignin surface hydroxyl group to form lignin urethanes moiety, thereby improving phase compatibility when it would mix with polyol and polyisocyanate during RPU foam synthesis (Scheme 1). This may improve dispersion of lignin in the crosslinking RPU system, which is expected to improve the cellular structure uniformity and the mechanical properties of RPU foams. 

In this article, surface functionalized lignin was used to substitute 0–40% of conventional petroleum-based polyol for the synthesis of bio-based RPU foams. As-synthesized surface functionalized lignin-based RPU (SFL-RPU) foam was compared to conventional petroleum-based RPU foams and to non-functionalized lignin-based RPU (L-RPU) foams in regard to its morphological, physicochemical, mechanical, and thermal properties. The SFL-RPU foams with up to 30% lignin-to polyol substitution ratio exhibited comparable thermal and mechanical properties when compared to conventional RPU foams. The specific compressive strength and modulus of the SFL-RPU foam surpassed the performance of L-RPU foams up to 47% and 45%, respectively. These findings along with decreased environmental cost supported by life cycle assessment suggest potential use of surface functionalized lignin as a sustainable core material replacing synthetic polyols in building applications.

## 2. Materials and Methods

### 2.1. Materials

A commercial USA softwood kraft lignin was used as lignin source for RPU foam preparation. The lignin sample was oven-dried at 105 °C for 24 h before it was used for the preparation of RPU foams. Commercial polyester polyol (R-23-015) and pMDI (A-23-015) were purchased from NCFI Polyurethanes (Mount Airy, NC, USA). Based on the specification provided by NCFI Polyurethanes, the polyol R-23-015 contains tertiary amine catalyst (<1%) and a physical blowing agent containing 1,1,1,3,3-Pentafluoropropane (<3%) and 1,1,1,3,3-Pentafluorobutane (<10%). The total amount of blowing agents in polyol R-23-015 measured by a gravimetric method in our laboratory was 9%. For lignin-based RPU foams, Dabco^®^ 33-LV (33 wt. % solution of triethylenediamine in dipropylene glycol, Sigma Aldrich, St. Louis, MO, USA) was used as a catalyst. 1,1,1,3,3-Pentafluorobutane (Fisher Scientific, Hampton, NH, USA) was used as a physical blowing agent for the preparation of lignin-based RPU foams. Other chemicals were purchased from Sigma-Aldrich as analytical grade and used as received.

### 2.2. Surface Functionalization of Lignin

Surface functionalization of lignin was carried out by pMDI under heat. Lignin, silicone oil, and excess pMDI were mixed into a 300-mL glass jar and stirred in a vacuum desiccator subjected to two cycles of vacuum (5 min at 250 Torr), followed by nitrogen gas (>99.99%) purging, to prepare a lignin-pMDI premix (ratios of the components listed in Table 1). The lignin-pMDI premix was then heated in a water bath at 80 °C for 1 h under stirring (800 rpm) to initiate surface functionalization. At the end of reaction, the mixture of surface functionalized lignin (SFL) and pMDI were termed as lignin-pMDI prepolymer, which was used immediately for SFL-RPU foam preparation.

### 2.3. Preparation of RPU Foams

Prior to the preparation of lignin-based RPU foams, 1,1,1,3,3-Pentafluorobutane and Dabco^®^ 33-LV were added into polyol R-23-015 in appropriate amounts (Table 1) in order to keep their ratios in pure R-23-015 and polyol/lignin mixtures constant. The polyol-premix was then sealed in a glass jar and shaken vigorously for 10 min, before conditioning for at least 24 h at 10 °C.

The RPU foams were prepared by adding polyol-premix into either lignin-pMDI premix or preprolymer, and stirring the combination at 2500 rpm for 10 s. Two premix mixtures were prepared for each formulation. The NCO index (isocyanate group equivalents/hydroxyl group equivalents × 100) between 150 and 160, as recommended by NCFI Polyurethanes, was used for all foams (Table 1). Each of the resultant mixtures were then immediately transferred into a 15 × 15 × 15 cm^3^ open mold and allowed to freely rise at room temperature.

All RPU foams were conditioned for at least 48 h under ambient conditions. The RPU foams prepared using SFL with the lignin-to-polyol substitution ratio of 10, 20, 30, and 40% were labeled as SFL-RPU10, SFL-RPU20, SFL-RPU30, and SFL-RPU40, respectively. The RPU foams prepared using non-functionalized lignin were labeled as L-RPU10, L-RPU20, L-RPU30, and L-RPU40, for the equivalent amount of lignin. The reference foam without lignin was labeled as RPU0. The foams were cut into samples of different sizes, depending on the type of analysis.

### 2.4. Lignin Structure Characterization

The ash content of kraft lignin was measured following American Society for Testing Materials (ASTM) D1102. In a typical example, three replicates of 1 g of lignin sample was calcined in a muffle furnace at 525 °C for at least 4 h.

Size exclusion chromatography (SEC) of acetylated lignin was used to determine molecular weight and polydispersity index of lignin. Acetylation was performed by suspending approximately 500 mg of oven-dry lignin sample in 20 mL of pyridine/acetic acid (*v*/*v* = 1:1) solution and stirring at room temperature. After 48 h, 12.5 mL of hydrochloric acid (0.1 mol/L) was gradually added to the cooled solution and kept at 0 °C in an ice bath in order to precipitate acetylated lignin. The precipitates were washed and neutralized with excessive deionized water, and then vacuum-dried at 40 °C overnight. SEC analysis of three lignin replicate samples was carried out by injecting 40 μL of the freshly filtered (0.45 μm PTFE syringe filter) acetylated lignin solution (1 mg/mL in tetrahydrofuran) into a PLgel 10 μm Mixed-D column (300 × 7.5 mm, Agilent Technologies, Inc., Santa Clara, CA, USA) equilibrated at 30 °C, and using tetrahydrofuran as an eluent at a flow rate of 1 mL/min. The analysis was performed on Thermo Finnigan Surveyor system coupled with Refractomax 520 refractive index (RI) detector. The calibration of the system was performed with polystyrene standards (Polymer Standards Servic-USA, Inc., Amherst, MA, USA) under the same conditions.

Lignin phosphitylation and phosphorus nuclear magnetic resonance (^31^P-NMR) analysis were performed to quantify hydroxyl functional groups in lignin. For phosphitylation, 20 mg of lignin sample replicates was dissolved in 0.5 mL of pyridine/CDCl_3_ solution (1.6:1 by *v*/*v*) containing 1 mg/mL of chromium acetylacetonate (relaxation agent) and 2 mg/mL of endo-*N*-hydroxy-5-norbornene-2,3-dicarboximide (internal standard), followed by adding 0.1 mL of 2-Chloro-1,3,2-dioxaphopholane (phosphitylation reagent) into the solvent. Quantitative ^31^P-NMR spectra were acquired after in situ phosphitylation of the lignin sample. ^31^P-NMR analysis of the lignin replicates was carried out using a Bruker Avance 400 MHz NMR spectrometer (Bruker, Inc., Billerica, MA, USA) housed in The Department of Chemistry, Mississippi State University. The NMR spectra were acquired at 25 °C using an inverse gated decoupling pulse sequence with a 90° pulse angle, 5 s pulse delay, and 128 scans. The same method was applied for determination of commercial polyol R-23-015 hydroxyl content.

### 2.5. Characterization of SFL

The rheological properties of 2 replicates of lignin-pMDI premix and prepolymer were determined using an AR 1500EX (TA Instruments, New Castle, DE, USA) rheometer equipped with a DIN concentric at an angular frequency of 0.1 rad/s were recoded for comparison. 

The SFL was extracted from lignin-pMDI prepolymer (20 wt. % lignin content) by washing the lignin-pMDI prepolymer with benzene to remove the unreacted pMDI. The Fourier-transform infrared spectroscopy (FTIR) spectra of five sub-samples of the SFL were recorded with Spectrum Two attenuated total reflection (ATR) spectrometer (PerkinElmer, Waltham, MA, USA) over the range of 720 to 4000 cm^−1^ (at a resolution of 2 cm^−1^) at the average of 10 scans. The spectra were baseline-corrected manually using Spectrum^®^ Quant software (PerkinElmer, Waltham, MA, USA). The baseline-corrected spectra were normalized over the total spectral area and analyzed by principal component analysis (PCA) using Unscrambler 10.3 X (CAMO Software Inc., Magnolia, TX, USA).

### 2.6. Characterization of RPU Foams

The apparent density of RPU foams was determined according to ASTM D1622-08. The cell morphology of the foams was analyzed by a scanning electron microscopy (SEM, JSM-6110 LV, JOEL, Akishima, Tokyo). A cross-section parallel to the foam rise direction was examined under the SEM. The samples (10 × 10 × 2 mm^3^) were sputter coated with 25 nm-thick platinum prior to SEM imaging. The average cell diameter was estimated from more than 200 measurements of each foam by using ImageJ software.

Thermal conductivity of foams was determined using a KD2 Pro (Decagon Devices Inc., Pullman, WA, USA) thermal conductivity meter. Ten measurements were conducted for one sample (50 × 50 × 100 mm^3^; 100 mm in rise direction) cut out of one foam of each sample group. The measurements were performed over 10 min, as suggested in previous reports [18].

To verify if the surface functionalization could increase the crosslinking density between SFL and pMDI, the RPU foams were extracted with 1,4-dioxane-water binary solutions (dioxane/water = 8/2, *w*/*w*), a good solvent of lignin. In the experiment, the foam was cut into three small pieces of approximately 8 × 8 × 8 mm^3^ and extracted with dioxane in a Soxhlet extractor for 24 h. The weight loss of RPU foams (of its original weight) after extraction was calculated for comparison. The mechanical properties of the foams were measured on eight replicates (50 × 50 × 25 mm^3^; 25 mm in rise direction), cut from two foam samples of each sample group. The measurements were carried out by Instron 3382 universal testing machine (Instron Corp., Norwood, MA, USA). Compressive strength (*σ*) and compressive modulus (*E*) in direction parallel to foam rise were measured and calculated according to ASTM D 1621-10 (crosshead movement rate at 2.5 mm/min). The specific compressive strength (*σ_ρ_*, compressive strength per unit density) and specific modulus (*E_ρ_*, compressive modulus per unit density) were calculated using the following equations:

(1) $$\sigma_{\rho }\left(kPa\;kg^{-1}\;m^{3}\right)=\frac{\sigma }{\rho }$$

(2) $$E_{\rho }\left(kPa\;kg^{-1}\;m^{3}\right)=\frac{E}{\rho }$$

where ρ is the density of foams.

In order to quantify the effects of lignin surface functionalization on the foam’s specific compressive properties, the following equations were used to calculate the changes of *σ_ρ_* and *E_ρ_* (Δ*σ_ρ_* and Δ*E_ρ_*) at a given lignin substitution ratio:

(3) $$\Delta \sigma_{\rho }\;\left(\%\right)=\frac{\sigma_{\rho }^{\mathrm{SFL}}-\sigma_{\rho }^{L}}{\sigma_{\rho }^{L}}\times 100$$

(4) $$\Delta E_{\rho }\;\left(\%\right)=\frac{E_{\rho }^{\mathrm{SFL}}-E_{\rho }^{L}}{E_{\rho }^{L}}\times 100$$

where $\sigma_{\rho }^{\mathrm{SFL}}$ is specific compressive strength of SFL-RPU foam (L represents lignin-to-polyol substitution ratio, 10 to 40%); $\sigma_{\rho }^{L}$ is specific compressive strength of L-RPU foam; $E_{\rho }^{\mathrm{SFL}}$ is specific compressive modulus of SFL-RPU foam (L represents lignin substitute ratio); $E_{\rho }^{L}$ is specific compressive modulus of L-RPU foam.

FTIR spectra of three sub-samples of the foams were recorded with Spectrum Two ATR spectrometer (PerkinElmer, Waltham, MA, USA) in the range of 450 to 4000 cm^−1^ (at a resolution of 2 cm^−1^) as an average of 10 scans. The spectra were baseline-corrected by “Data Tune-up” transformation option, and normalized with respect to aromatic rings band (at 1510 cm^−1^) using Spectrum^®^ Quant software. The intensity ratio of N=C=O band to C=O band (I_NCO_/I_CO_) was calculated based on the normalized spectra. 

Thermogravimetric analyses (TGA) of the foams were performed by 50 H thermo-gravimetric analyzer (TA Instruments, New Castle, DE, USA). Two replicates (~5 mg each) of each sample type were heated from 50 °C to 750 °C at a ramping rate of 10 °C/min in flowing nitrogen (99.99%, 100 mL/min) atmosphere under ambient pressure. The differences between the replicates were negligible in comparison to the differences between the samples.

The foam density, cell size, thermal conductivity, mechanical properties, and I_NCO_/I_CO_ were analyzed and compared by analysis of variance (ANOVA) using SAS software (SAS Institute, Cary, NC, USA). A one-factor ANOVA was performed to analyze significance of differences among the sample groups. All statistical analyses were performed at a significance level of 0.05.

### 2.7. Life Cycle Assessment

The life cycle assessment (LCA) was performed with openLCA 1.6.3 (GreenDelta, Berlin, Germany). The ecoinvent database 2.2 was used to model the life cycle inventory concerning the process flows. The kraft lignin lifecycle data was downloaded from the USDA LCA Commons public domain from previous research [24]. Tables S1–S4 shows the inventory data from the ecoinvent database, which was slightly modified so that equivalent comparisons of the processes could be made. Table S5 shows the inventory data from the kraft lignin lifecycle dataset [25]. Electricity needed to produce the polyurethane and waste heat generation were ignored involving the polyurethane formation. The tool for reduction and assessment of chemicals and other environmental impacts (TRACI—from the EPA) life cycle impact assessment (LCIA) protocol used to determine the effects of the flows used in this study.

## 3. Results and Discussion

### 3.1. Lignin Properties

Ash content, molecular weight, and hydroxyl value of lignin are important parameters as they influence overall properties of the resultant RPU foams. The hydroxyl content and molecular weight of lignin were estimated from ^31^P-NMR spectrum (shown in Figure S1 in Supporting Information) and SEC analysis, and the results summarized in Table 2. The comparable hydroxyl contents of lignin (5.24 mmol/g) and the commercial polyol (5.34 mmol/g) suggest potential of lignin to be a good alternative for polyols in the case of preparation of RPU foams. However, the higher content of phenolic hydroxyl groups in lignin (58% of total OH) in comparison to commercial polyol (2.1% of total OH) indicates lower reactivity of lignin.

### 3.2. Rheological Behavior of Lignin-pMDI Premix and Prepolymer

**Figure 1:**
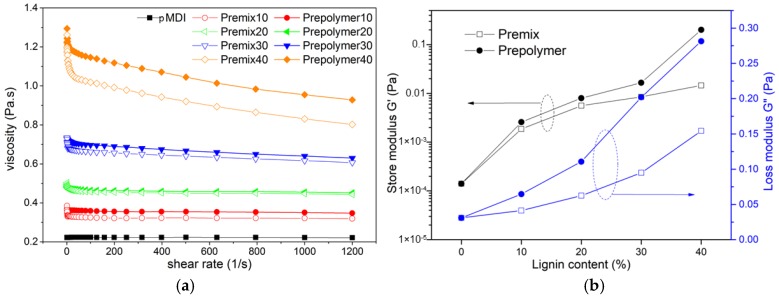
(**a**) Viscosity vs. shear rate curves of raw polyisocyanate methyl diphenyl diisocyanate (pMDI) resin (black), lignin-pMDI premix (hollow symbols), and lignin-pMDI prepolymer (solid symbols) containing 10–40% lignin; (**b**) storage (G′) and loss (G″) modulus of various lignin-pMDI premixes and the prepolymers at an angular frequency of 0.1 rad/s.

Figure 1a shows the viscosity of the lignin-pMDI premix and the prepolymer with respect to shear rate. The viscosity is strongly dependent on the lignin content in the mixture. The viscosity of mixture at a shear rate of 10 s^−1^ increased from 0.2 to 1.2 Pa·s upon addition of lignin in the amount of 40%, probably due to the filler effects of lignin [26]. At the same lignin content, the lignin-pMDI prepolymer exhibited apparently higher viscosity than lignin-pMDI premix, which suggests lignin was successfully functionalized by pMDI and formed lignin-pMDI macromolecules. Figure 1b shows the storage (G′) and loss (G″) modulus of various lignin-pMDI premixes and the prepolymers at an angular frequency of 0.1 rad/s. Both G′ and G″ were found increased with the increase of lignin content. The increase of G′ and G″ at high lignin content could be related to the filler effects of lignin particles and the interaction of lignin to pMDI [27]. At the same lignin content, the lignin-pMDI prepolymer exhibited higher G′ and G″ than lignin-pMDI premix, which also suggests the surface functionalization of lignin and the formation of cross-linked lignin-pMDI macromolecules in lignin-pMDI prepolymers.

**Figure 2:**
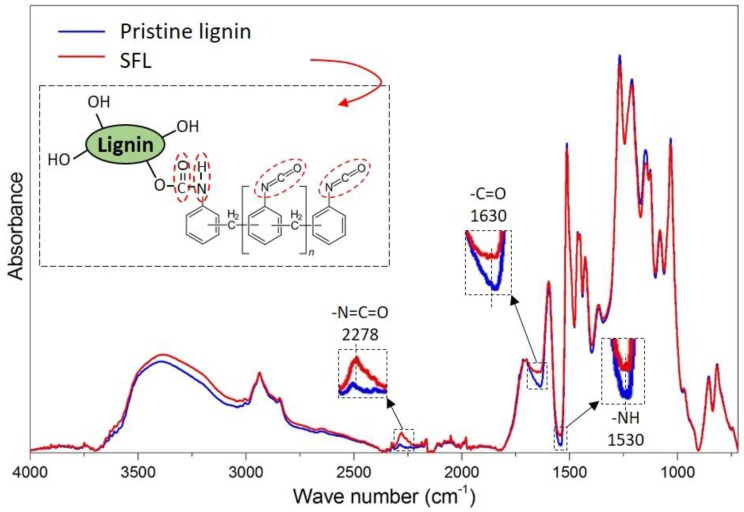
Baseline corrected and normalized Fourier-transform infrared spectroscopy (FTIR) spectra of pristine lignin and SFL.

Our hypothesis was that the isocyanate groups in pMDI react with lignin surface hydroxyl groups to form urethane bonds on lignin surface. To verify this hypothesis, the extracted SFL from lignin-pMDI prepolymer was investigated by FTIR and the data was analyzed by PCA. Figure 2 shows the normalized FTIR spectra of pristine lignin and the SFL. The SFL shows higher vibration intensity of the isocyanate (–N=C=O, 2278 cm^−1^), carbonyl (–C=O, 1630 cm^−1^) and amine –NH (1530 cm^−1^) bands [28]. The score plot (Supporting Figure S2a) of FTIR spectra shows clusters of the two sample groups according to PC1, where pristine lignin has negative loading on PC1 and the SFL have positive loading on PC1. The loading plot (Supporting Figure S2b) shows positive values at absorption bands of –C=O (1630 cm^−1^), –N=C=O (2278 cm^−1^), and –NH (1530 cm^−1^), confirming presence of the isocyanate groups in SFL. The shift of N=C=O and NH from 2242 and 1522 cm^−1^ in MDI (Supporting Figure S3) to 2278 and 1530 cm^−1^ in SFL suggests and the formation of urethane bonds between lignin and pMDI.

### 3.3. Physical Properties of RPU Foams

**Figure 3:**
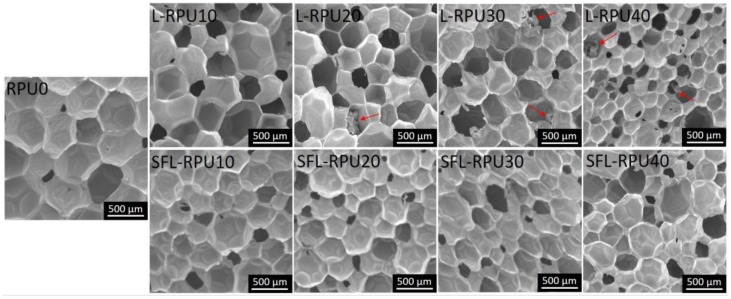
Scanning electron microscopy (SEM) images of L-RPU (**top row**) and SFL-RPU (**bottom row**) foams made with different amounts (0–40%) of lignin.

The photographs and the SEM images of RPU foams are shown in Figure 3 and Supporting Figure S4. RPU foams became darker with increase in lignin substitution ratio, which can be attributed to the light-absorbing property of lignin. The dark color of lignin-based RPU foams is acceptable because the foams are used as a core material in building applications. The number of small particles (dark spots in the images) and agglomerates observed in the lignin-based RPU foams, increased with the rise of lignin content. RPU foams were also scanned by SEM to determine homogeneity of the cellular structure. RPU foams with up to 20 wt. % lignin replacement ratio revealed homogeneous cell structure (Figure 3). With the increase in lignin content, the cell shape became inhomogeneous and less regular, with higher number of defective and distorted cells. The SFL-RPU foams displayed more defective cells and lignin agglomerates (indicated by red arrows) than L-RPU foams, which indicates that the lignin surface functionalization can increase the isotropy of lignin-based foam cellular structure.

Table 3 lists the properties of the lignin-based (L-RPU and SFL-RPU) and the reference (RPU0) foams including cell diameter, apparent density, and thermal conductivity. The average cell diameter decreased significantly with the increase of lignin content. The apparent density of RPU foams increased significantly with the increase of lignin substitution ratio.

At the 40% of lignin substitution ratio, the density increased by 58% and 25% in L-RPU40 and SFL-RPU40 foams, respectively. For the same foaming parameters (i.e., NCO index and amount of blowing agent), the higher foam density indicates the poor foaming process, which can be attributed to lignin particle agglomeration. Thus, the lower density of SFL-RPU40 than L-RPU40 echoes with the SEM observations and confirms our hypothesis that surface functionalization improves dispersion of lignin in the crosslinking RPU system. The increase in lignin substitution ratio resulted in higher density and smaller cell diameter of foams was also reported by Luo et al. and Camila et al. [17,29]. This can be attributed to three reasons. Firstly, the addition of lignin increases the mixture viscosity (Figure 1) which restrains the expansion of the pores with smaller diameters, and reduces the free rise volume of foams [17,29,30]. Secondly, lignin has lower reactivity than a commercial polyol and the gelation reaction rate is lower, which allows more gases to escape from the foam structure, consequently decreasing the pore size and free rise volume [22]. Lastly, lignin powder could act as a nucleation site to facilitate the nucleation of bubbles and lead to a smaller cell size [18].

Thermal conductivity is an important factor for the application of RPU foams as building materials and it is closely related to the foam density and cell diameter. Generally, the thermal conductivity value is directly proportional to the foam apparent density and inversely proportional to the cell size. The thermal conductivity values of lignin-based RPU foams meet the thermal conductivity requirements for structural insulation materials specified in ASTM E1730. Compared with RPU0, the lignin-based RPU foams with up to 30% lignin substitution ratio showed slight reduction in thermal conductivity, which can be attributed to the cell size reduction. However, the thermal conductivity of L-RPU40 was higher than RPU0, which may be due to high density and defective cells. Therefore, the use of lignin in the substitution ratio up to 30% seems beneficial for increase of thermal insulation properties of as-synthesized RPU foams.

**Figure 4:**
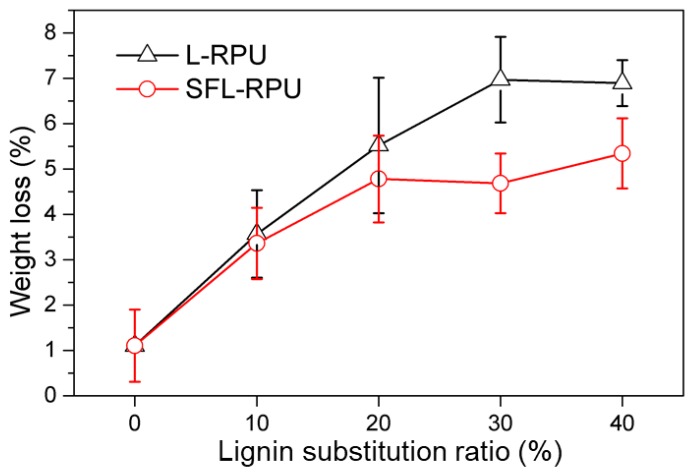
Weight loss of PU0, SFL-RPU and L-RPU foams after extraction.

To verify if the surface functionalization could increase the crosslinking density between SFL and pMDI, the RPU foams were extracted with a dioxane-water solution. The weight loss of RPU foams after extraction was calculated and shown in Figure 4. The reference foam (RPU0) is found to have the lowest weight loss of 1.10% after the extraction. With the increase of lignin substitution ratio from 10% to 40%, the weight loss increased from 3.57 to 6.89% and 3.36 to 5.34% for L-RPU and SFL-RPU foams, respectively. At the same lignin substitution ratio, the SFL-RPU foams exhibit lower weight loss than that of L-RPU, which confirms the surface functionalization increased the crosslinking density between SFL and pMDI in the foams.

### 3.4. Mechanical Properties of RPU Foams

The RPU foams obtained in this study showed average compressive strength of 136 to 205 kPa (Table S6), which meets the compressive properties requirements for insulation materials for wall panel applications specified in ANSI/APA PRS 610.1-2013. It is worthwhile to mention that the compressive strength (*σ*) and modulus (*E*) of the RPU foam prepared with 30% functionalized lignin (SFL-RPU30) are above 200 kPa, which is higher than what has been reported by Camila et al. (<100 kPa) and Xue et al. (140 kPa) [17,20].

**Figure 5:**
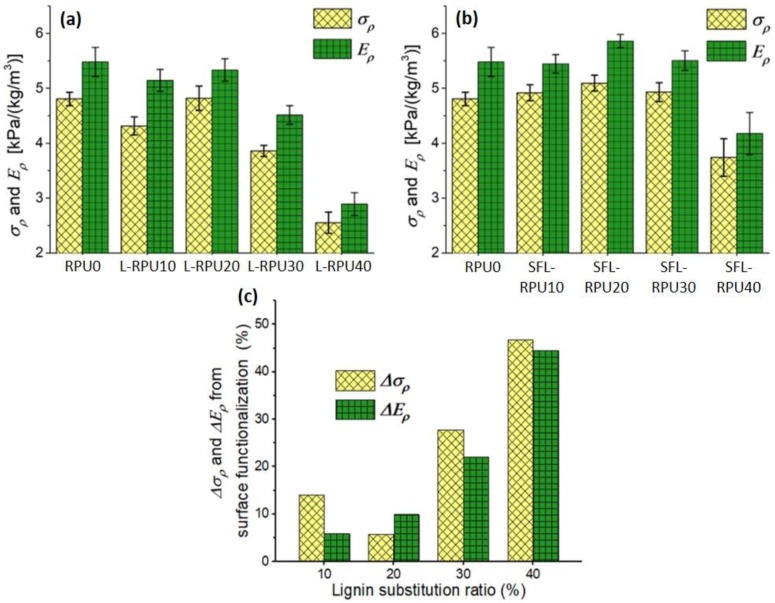
Specific compressive strength *(σ_ρ_*) and modulus (*E_ρ_*) of L-RPU (**a**) and SFL-RPU (**b**) containing different lignin substitution ratios, and the change of *σ_ρ_* and *E_ρ_* (Δ*σ_ρ_* and Δ*E_ρ_*) of lignin-containing RPU foams at given lignin substitution ratios (**c**).

It is well known that the mechanical strength of RPU foams is proportional to foam apparent density [17,31]. As described before, the foam apparent density increased with the increase of lignin substitution ratio. Thus, to exclude the effect of density on the *σ* and *E* of the foams, the specific compressive strength (*σ_ρ_*, compressive strength per unit density) and specific modulus (*E_ρ_*, compressive modulus per unit density) were calculated (Table S6). All RPU foams prepared without surface functionalization (L-RPU10 to L-RPU40) showed lower *σ_ρ_* and *E_ρ_* (Table S6 and Figure 5a) than the reference (RPU0), and in the case of L-RPU30 and L-RPU40 foams, the differences were significant. This is attributed to the lower reactivity and poor dispersion of lignin than substituted polyols that inevitably decreased the density of cross-linked polyurethane chains in the foams. Additionally, the introduction of lignin aggregates would increase the number of defective foam cells and decrease the uniformity of the foam cellular structure, as shown in SEM images in Figure 3. By contrast, the *σ_ρ_* and *E_ρ_* of the SFL-RPU foams with up to the 30% substitution ratio showed slightly higher values than that of the reference RPU0 (Table S6 and Figure 5b), although the differences were not significant. As the lignin ratio increased from 0 to 20%, the *σ_ρ_* and *E_ρ_* of RPU foams showed a trend of increasing values. Further increase in lignin content leads a reduction in both values.

For the RPU foams containing lignin substitute of up to 20%, there was no significant difference between L-RPU and SFL-RPU foams. However, the foams containing lignin substitute greater than 30%, the SFL-RPU foams showed significantly higher *σ_ρ_* and *E_ρ_* (Table S6) than L-RPU foams. As shown in Figure 5c, the change in specific compressive strength, Δ*σ_ρ_* was 28% and 47%, and the change in specific modulus, Δ*E_ρ_*, was 22% and 45% for the foams with lignin substitute of 30% and 40%, respectively. We believe the improvement in *σ_ρ_* and *E_ρ_* of the RPU foams prepared using SFL is attributed to the increase in crosslinking density of RPU foams and enhanced compatibility of lignin and polyisocyanate, as well as the good dispersion of lignin in foam cellular structure, as indicated by the SEM images in Figure 3.

### 3.5. FTIR Analysis of RPU Foams

**Figure 6:**
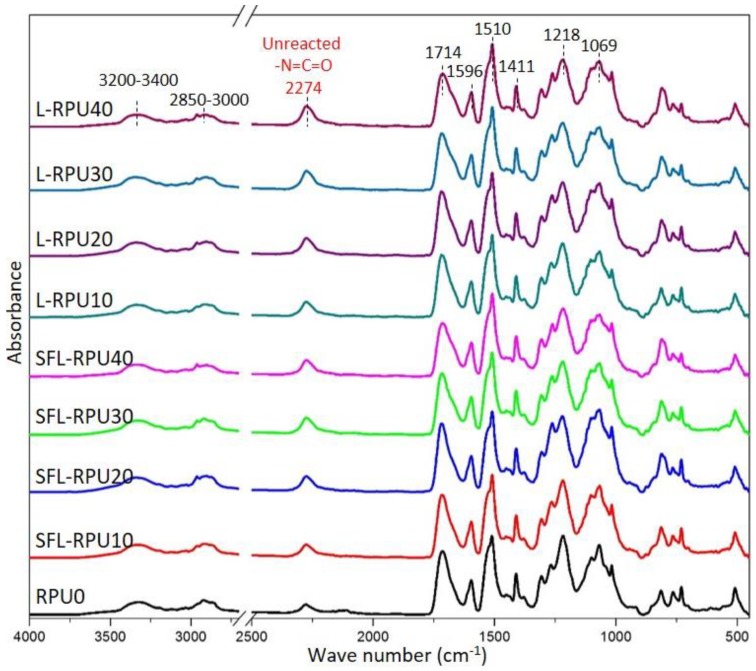
FTIR spectra RPU foams containing 0–40% lignin.

Figure 6 shows the baseline corrected and normalized FTIR spectra of the all RPU foams. The urethane-related linkages such as N–H (3200–3400 cm^−1^), C=O (1714 cm^−1^), C–O (1069 cm^−1^), and C–N (1218 cm^−1^) peaks can be observed in all spectra [10,18]. The peaks at 1510 and 1596 cm^−1^ can be attributed to the vibration bands of aromatic rings originating from pMDI, polyester polyol, and lignin raw materials [18]. Additionally, the isocyanate peak at 2274 cm^−1^, observed in all the RPU foams, is attributed to the residual isocyanate groups. This residual isocyanate group is expected to exist in the foams since the amount of MDI available for the reaction is greater than stoichiometric amount for the polyols added (initial NCO index was ~155).

During the formation of RPU foams, isocyanate groups in pMDI undergo urethane formation with polyol and lignin hydroxyls (Scheme 1), and as a result, the intensity of N=C=O (I_NCO_) decreases and intensity of C=O (I_CO_) increases [32]. Thus, the amount of residual, i.e., unreacted pMDI in the foams prepared from different lignin amounts can be estimated through the peak intensity ratios (I_NCO_/I_CO_) shown in Table S7. At the given lignin substitution ratio, the I_NCO_/I_CO_ of SFL-RPU foams is always lower than that of L-RPU, which indicates improved reactivity of SFL toward urethane formation in the foams and echoes with the overall improved mechanical properties of the SFL-RPU foams.

### 3.6. Thermal Stability of RPU Foams

**Figure 7:**
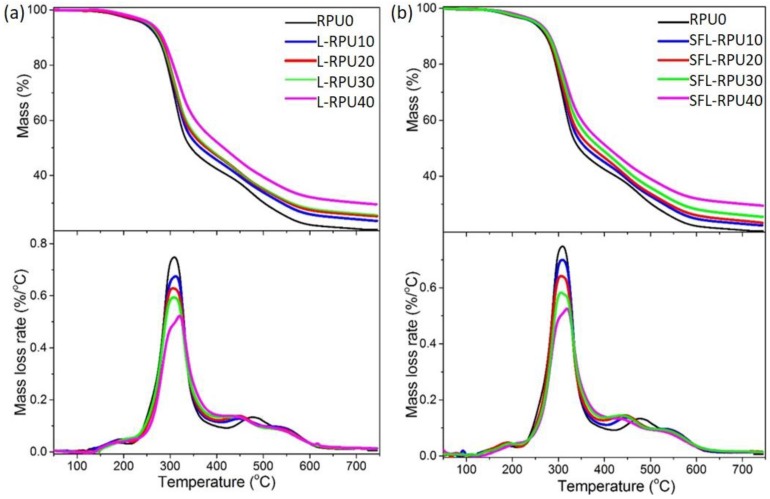
Thermogravimetry (TG) and the maximum derivative thermogravimetric (DTG) curves of L-RPU (**a**) and SFL-RPU (**b**) foams containing 0–40% lignin.

Thermogravimetric (TG) curves of the RPU foams are displayed in Figure 7. All the foam samples degraded in one broad temperature range of 150–650 °C (with the maximum derivative thermogravimetric (DTG) peak at ~310 °C). The thermal degradation temperatures and char yield are presented in Table S8. The onset of thermal degradation temperature (T_5%_, defined as the temperature at 5% mass loss) shifted to higher temperature as the lignin content increased. This improved thermal stability is possibly a result of the highly cross-linked thermostable segments produced from the interactions between lignin and the foam matrix [12,31]. The maximum mass loss rate (DTG-max) also showed a decreasing trend as lignin content in the foams increased. This is mainly due to the higher aromatic density in the lignin-based RPU foam network [12]. The char yield increased with the increase of lignin content in foams, which is also due to the high aromatic density and thermostable nature of lignin. These properties confirmed that use of lignin is beneficial for production of thermal insulating foams.

### 3.7. Life Cycle Assessment

**Figure 8:**
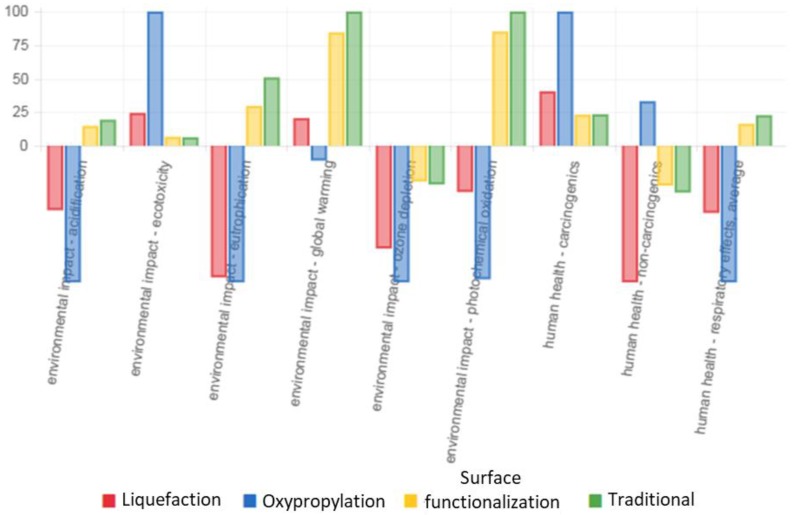
Life Cycle Assessment comparison of liquefaction, oxypropylation, surface functionalization, and traditional processing to manufacture polyurethane. For each indicator, the maximum result is set to 100% and the results of the other variants are displayed in relation to this result.

Although the use of lignin as a substitute for synthetic polyols in the production of RPU foams is not new, chemical modification of lignin to polyol such as liquefaction and oxypropylation usually involves harmful chemicals including H_2_SO_4_ and KOH, which leaves a substantial environmental footprint. In contrast, the proposed lignin surface functionalization with isocyanates is free of those toxic substances. A cradle-to-grave analysis was performed to quantitatively assess environmental impacts associated with the production of RPU foams with using kraft lignin. As summarized in Figure 8, the LCA results showed that the lignin surface functionalization could surpass traditional manufacturing of polyurethane in both environmental and human health areas of concern such as acidification, eutrophication, global warming, photochemical oxidation, carcinogenics, and respiratory effects. The lignin surface functionalization had a lesser effect on the environment concerning ecotoxicity when compared to both the liquefaction and oxypropylation processes. The lignin surface functionalization also had a less of an effect on human health concerning carcinogen creation when compared with the liquefaction and oxyproplyation processes and less of an effect on non-carcinogens when compared with the oxypropylation process. Details outlining the measurements of the units concerning the impact and the model output stipulations were shown in Tables S9 and S10, respectively.

## 4. Conclusions

In summary, a simple lignin surface functionalization with polyisocyanate poses an attractive strategy for the development of high-lignin-content RPU foams for construction and structural applications. Lignin-based RPU foams with as high as 30% of lignin substitution of petroleum-based polyol exhibited comparable thermal and mechanical properties to conventional RPU foams. The mechanical performance of the SFL-RPU foams outperformed the L-RPU foams by up to an ~50% increase in compressive strength. Lignin surface characteristics had changed during the surface functionalization due to the conversion of lignin hydroxyl groups into lignin urethanes moiety, thereby enabling good lignin dispersion and reactivity, which is beneficial for the enhancement of RPU foam mechanical properties.

## Figures and Tables

**Table 1:** Amounts of components used for the preparation of RPU foams.

Lignin Substitution Ratio (%)	Lignin-pMDI Premix (g)			Polyol Premix (g)			NCO Index
	Lignin	pMDI	Silicone Oil *	Polyol R-23-015	Dabco 33-LV *	1,1,1,3,3-Pentafluorobutane *	
0	0	51.5	0	50	0	0	157
10	5	51.5	0.25	45	0.025	0.45	156
20	10	51.5	0.50	40	0.050	0.90	154
30	15	51.5	0.75	35	0.075	1.35	153
40	20	51.5	1.00	30	0.100	1.80	152

Note: The amounts of silicone oil, Dabco^®^ 33-LV, and 1,1,1,3,3-Pentafluorobutane are 5%, 0.5%, and 9% in terms of lignin weight, respectively.

**Table 2:** Composition of lignin and polyol R-23-015.

Samples	Ash (wt. %)	Mn (g/mol)	Mw (g/mol)	Mw/Mn	Al-OH (mmol/g)	Ph-OH (mmol/g)	COOH (mmol/g)	Total OH (mmol/g)
Lignin	1.65 ± 0.04	816	3374	4.13	1.91 ± 0.12	3.04 ± 0.21	0.29 ± 0.02	5.24 ± 0.35
Polyol	0	-	-	-	5.21 ± 0.25	0.11 ± 0.01	0.02 ± 0.00	5.34 ± 0.25

**Table 3:** Cell diameter, density, and thermal conductivity of RPU foams containing 0–40% lignin.

Samples	Cell Diameter (μm)	Density (kg/m^3^)	Thermal Conductivity (mW·m^−1^·K^−1^)
RPU0	588 ± 85	34.6 ± 1.8	24.1 ± 0.5
L-RPU10	548 ± 154	33.9 ± 2.3	23.8 ± 0.4
L-RPU20	448 ± 135	36.2 ± 1.9	24.0 ± 0.4
L-RPU30	393 ± 93	41.7 ± 1.7	23.9 ± 0.3
L-RPU40	255 ± 67	53.3 ± 3.3	25.0 ± 0.7
SFL-RPU10	419 ± 98	35.5 ± 1.3	24.0 ± 0.6
SFL-RPU20	347 ± 132	38.5 ± 1.8	23.6 ± 0.5
SFL-RPU30	305 ± 135	41.6 ± 1.8	23.3 ± 0.5
SFL-RPU40	304 ± 99	42.2 ± 1.6	24.2 ± 0.4

## Supplementary Materials

The supplementary materials are available online at http://www.mdpi.com/2073-4360/10/7/706/s1.
