# Supplementary material for: Effects of Surface Functionalization of Lignin on Synthesis and Properties of Rigid Bio-Based Polyurethanes Foams
Source: Polymers (Basel). 2018 Jun 26;10(7):706. doi: 10.3390/polym10070706 (PMC6404063; doi:10.3390/polym10070706)
Supplement: Supplementary file 1 [file polymers-10-00706-s001.pdf]

Supporting Information

Effects of Surface Functionalization of Lignin on Synthesis and Properties of Rigid Bio-based Polyurethanes Foams

Xuefeng Zhang*, Dragica Jeremic, Yunsang Kim, Jason Street, Rubin Shmulsky*
Department of Sustainable Bioproducts, Mississippi State University, Mississippi State, MS 39762, USA

Contents:

10 Pages, including 10 tables and 4 figures.

1. Inventory data for life cycle assessment (LCA) ………………………….S2-S4

2. Characterization of lignin and polyol …………………………….…..S5-S8

3. LCA units and model output ……………………………………….…..S9

Inventory data for life cycle assessment (LCA)

Table S1. Flows of traditional polyurethane creation.

**Flow input**	**Category**	**Amount**	**Unit**
disposal, polyurethane, 0.2% water, to municipal incineration - CH	waste management/municipal incineration	0.02	kg
methylene diphenyl diisocyanate, at plant - RER	plastics/monomers	0.5	kg
pentane, at plant - RER	chemicals/organics	0.054	kg
polyols, at plant - RER	plastics/monomers	0.5	kg
**Flow Output**	**Category**	**Amount**	**Unit**
Pentane	Emission to air/high population density	0.003	kg
polyurethane, rigid foam, at plant - RER	plastics/polymers	1	kg

Table S2. Flows of functionalized lignin-based polyurethane creation.

**Flow input**	**Category**	**Amount**	**Unit**
disposal, polyurethane, 0.2% water, to municipal incineration - CH	waste management/municipal incineration	0.02	kg
kraft lignin (Bernier)	Agriculture, forestry	0.15	kg
methylene diphenyl diisocyanate, at plant - RER	plastics/monomers	0.5	kg
pentane, at plant - RER	chemicals/organics	0.054	kg
polyols, at plant - RER	plastics/monomers	0.35	kg
**Flow Output**	**Category**	**Amount**	**Unit**
Pentane	Emission to air/high population density	0.003	kg
polyurethane, rigid foam, at plant - RER	plastics/polymers	1	kg

Table S3. Flows of liquefaction polyurethane creation.

**Flow input**	**Category**	**Amount**	**Unit**
disposal, polyurethane, 0.2% water, to municipal incineration - CH	waste management/municipal incineration	0.02	kg
kraft lignin (Bernier)	Agriculture, forestry	0.15	kg
methylene diphenyl diisocyanate, at plant - RER	plastics/monomers	0.5	kg
pentane, at plant - RER	chemicals/organics	0.054	kg
sulphuric acid, at plant	ecoinvent 2.2	0.015	kg
glycerine, from epichlorohydrin, at plant - RER	chemicals/organics	0.07	kg
ethylene oxide, at plant –RER	chemicals/organics	0.27933	kg
ethylene glycol, at plant - RER	chemicals/organics	0.00098	kg
**Flow Output**	**Category**	**Amount**	**Unit**
Pentane	Emission to air/high population density	0.003	kg
polyurethane, rigid foam, at plant - RER	plastics/polymers	1	kg

Polyethylene glycol was modeled using the following chemical reaction of ethylene glycol and ethylene oxide with a chain length of 400 (n=400)

HOCH2CH2OH + n(CH2CH2O) → HO(CH2CH2O)$_{n+1}$H

Table S4. Flows of oxypropylation polyurethane creation.

Flow input	Category	Amount	Unit
disposal, polyurethane, 0.2% water, to municipal incineration - CH	waste management/municipal incineration	0.02	kg
kraft lignin (Bernier)	Agriculture, forestry	0.15	kg
methylene diphenyl diisocyanate, at plant - RER	plastics/monomers	0.5	kg
pentane, at plant - RER	chemicals/organics	0.054	kg
potassium hydroxide, at regional storage - RER	chemicals/inorganics	0.015	kg
propylene oxide, liquid, at plant - RER	chemicals/organics	0.35	kg
**Flow Output**	**Category**	**Amount**	**Unit**
Pentane	Emission to air/high population density	0.003	kg
polyurethane, rigid foam, at plant - RER	plastics/polymers	1	kg

Table S5. Flows of kraft lignin creation (Bernier) [S1].

Flow input	Category	Amount	Unit
carbon dioxide, at plant/RER U	ecoinvent2.2	0.3	kg
electricity, medium voltage, at grid - US	ecoinvent2.2	0.01	kWh
natural gas, burned in boiler modulating >100kW	ecoinvent2.2	31.5	MJ
lime, hydrated, packed, at plant	ecoinvent2.2	0.23	kg
sodium hydroxide, 50% in H_2O, production mix, at plant	ecoinvent2.2	1.07	kg
sulphuric acid, liquid, at plant	ecoinvent2.2	0.23	kg
transport, lorry 16-32t, EURO4	ecoinvent2.2	0.934	t×km
Water, process, unspecified natural origin	water/unspecified	0.00485	m^3
**Flow Output**	**Category**	**Amount**	**Unit**
kraft lignin (Bernier)	Agriculture, forestry	1	kg

Characterization results

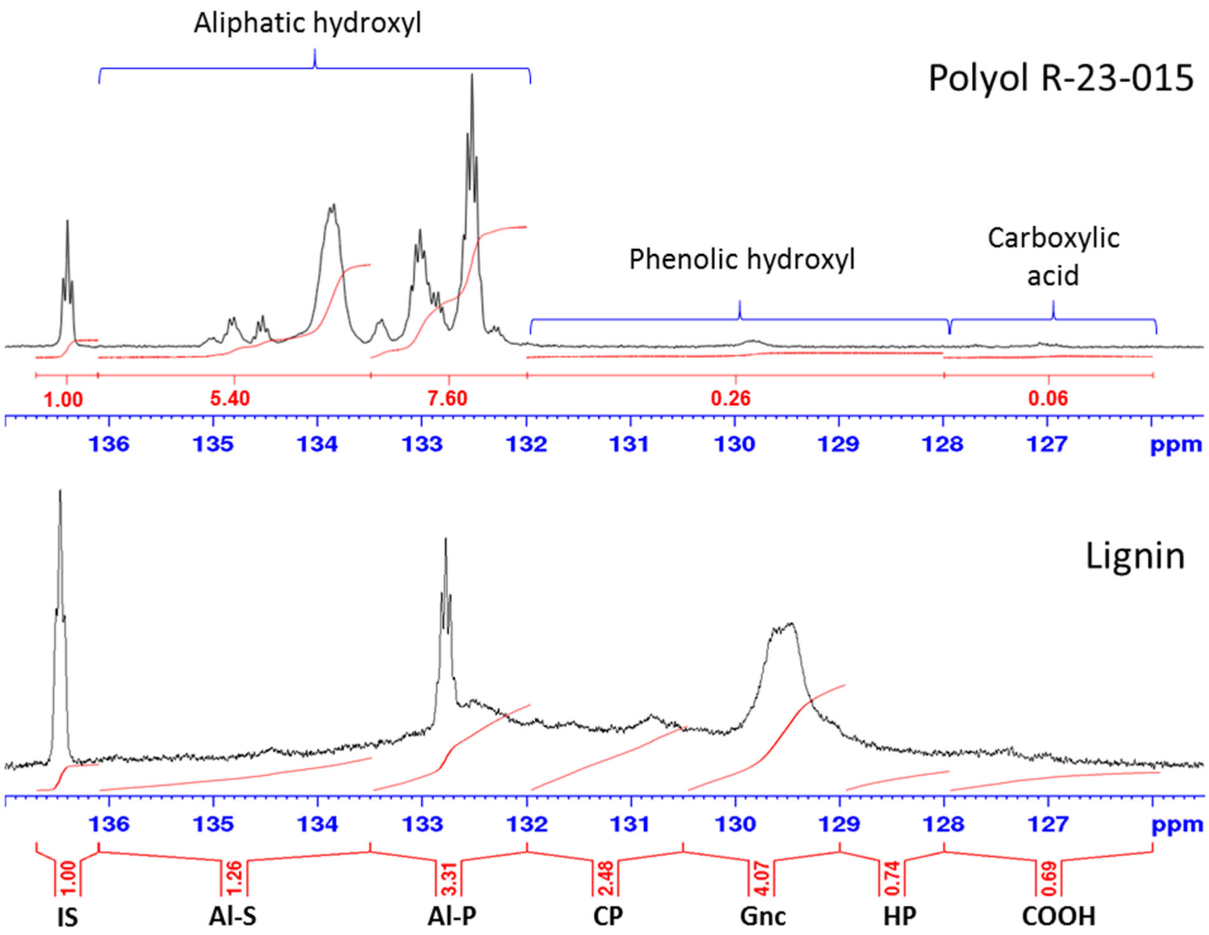

Figure S1. ^{31}P-NMR spectra of the polyol R-23-015 and lignin. IS, internal standard (136.7-136.1 ppm); Al-S, secondary aliphatic OH (136.1-133.5 ppm); Al-P, primary aliphatic OH (133.5-132.0 ppm); CP, condensed phenolic OH (132-130.5 ppm); Gnc, non-condensed guaiacyl (130.5-129 ppm); HP, hydroxyphenyl OH (129-128 ppm); COOH, carboxylic acid (128-126 ppm).

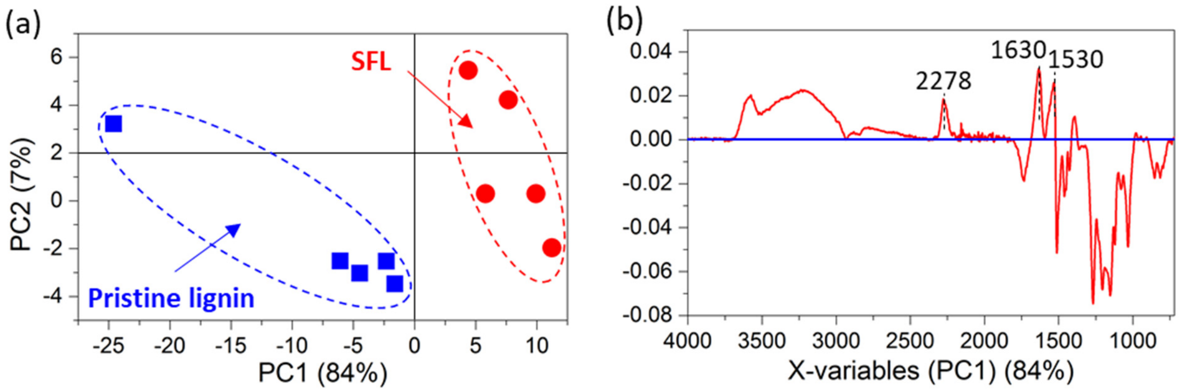

Figure S2. (a) score plot of PCA model (PC1 vs. PC2) and (b) loading plot of PC1 of PCA model.

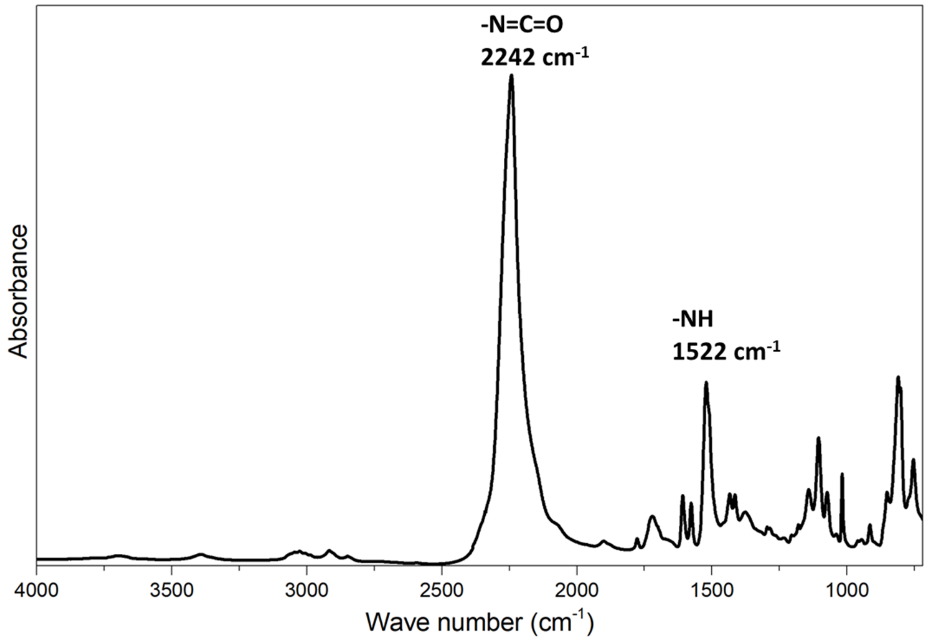

Figure S3. FTIR spectrum of pMDI (A-23-015).

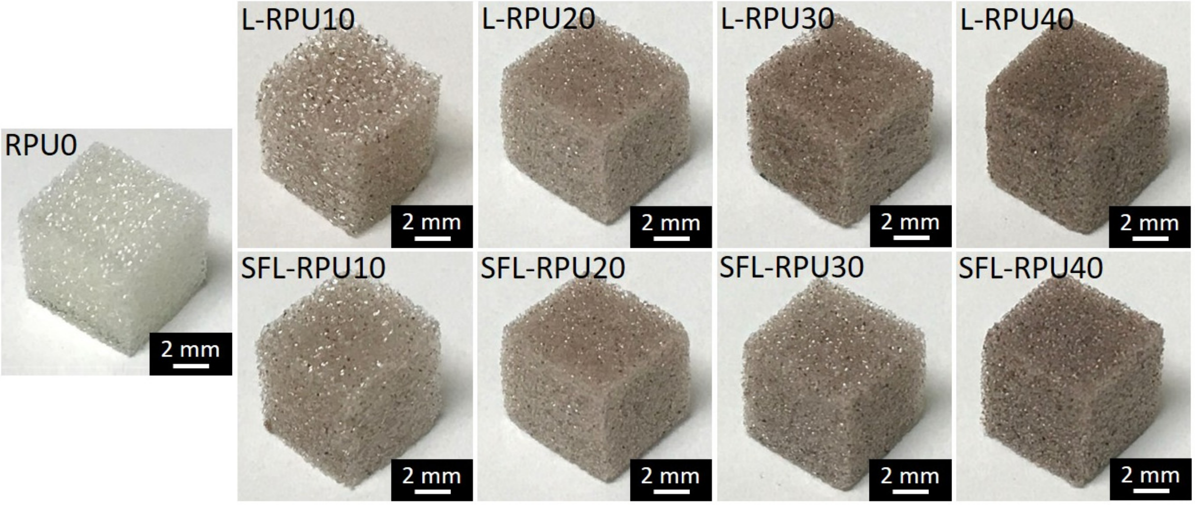

Figure S4. Photographs of RPU foams made with different amounts (0-40%) of lignin: L-RPU (top row) and SFL-RPU (bottom row).

Table S6. Compressive properties of RPU foams containing 0-40% lignin.

**Samples**	**Compressive strength** **(σ, kPa)**	**Compressive modulus** **(E, kPa)**	**Specific compressive strength** **[σ_ρ, kPa/(kg/m^3)]**	**Specific compressive modulus** **[E_ρ, kPa/(kg/m^3)]**
**RPU0**	166.3±7.7	189.5±26.0	4.8±0.4	5.5±0.8
**L-RPU10**	146.2±16.5	174.3±21.1	4.3±0.5	5.1±0.7
**L-RPU20**	174.4±32.8	193.0±31.7	4.8±0.7	5.3±0.7
**L-RPU30**	160.9±19.2	188.1±29.5	3.9±0.3	4.5±0.6
**L-RPU40**	135.9±33.5	153.9±35.6	2.5±0.5	2.9±0.6
**SFL-RPU10**	174.6±14.3	193.2±18.2	4.9±0.4	5.4±0.5
**SFL-RPU20**	196.1±19.7	225.5±14.8	5.1±0.4	5.9±0.4
**SFL-RPU30**	205.1±26.5	228.9±27.9	4.9±0.6	5.5±0.6
**SFL-RPU40**	157.8±51.8	176.1±57.8	3.7±1.1	4.2±1.3

Table S7. The intensity ratio of N=C=O band to C=O band (I_{NCO}/I_{CO}) of RPU foams containing 0-40% lignin.

**Samples**	**I_{NCO}/I_{CO}**
**RPU0**	0.17±0.04
**L-RPU10**	0.27±0.04
**L-RPU20**	0.30±0.05
**L-RPU30**	0.36±0.04
**L-RPU40**	0.41±0.05
**SFL-RPU10**	0.21±0.02
**SFL-RPU20**	0.25±0.03
**SFL-RPU30**	0.29±0.03
**SFL-RPU40**	0.32±0.06

Table S8. TGA results ($T_{5\%}$, DTG-max temperatures, and char yield at 750°C) of RPU foams.

**Sample**	**$T_{5\%}$ (°C)**	**DTG-max (°C)**	**Char yield at 750°C (wt%)**
**RPU0**	240.3	0.757	20.3
**L-RPU10**	245.9	0.698	22.7
**L-RPU20**	252.6	0.609	25.2
**L-RPU30**	251.8	0.578	26.2
**L-RPU40**	258.9	0.514	28.4
**SFL-RPU10**	245.2	0.720	22.1
**SFL-RPU20**	246.4	0.661	23.1
**SFL-RPU30**	247.6	0.585	25.2
**SFL-RPU40**	253.9	0.517	29.1

LCA units and model output

Table S9. LCIA Categories and Units

**Impact category**	**Unit**
environmental impact - acidification	moles of H+-Eq
environmental impact - ecotoxicity	kg 2,4-D-Eq
environmental impact - eutrophication	kg N
environmental impact - global warming	kg CO^2-Eq
environmental impact - ozone depletion	kg CFC-11-Eq
environmental impact - photochemical oxidation	kg NO_x-Eq
human health - carcinogenics	kg benzene-Eq
human health - non-carcinogenics	kg toluene-Eq
human health - respiratory effects, average	kg PM2.5-Eq

Table S10. Desired model output stipulations.

**Name**	**Product system**	**Flow**	**Amount**	**Unit**
Liquefaction	Liquefaction Method, at plant	polyurethane, rigid foam, at plant - RER	1	kg
Oxypropylation	Oxypropylation Method, at plant	polyurethane, rigid foam, at plant - RER	1	kg
Surface Functionalization	Surface Functionalization Method, at plant	polyurethane, rigid foam, at plant - RER	1	kg
Traditional	polyurethane, rigid foam, at plant (modified)	polyurethane, rigid foam, at plant - RER	1	kg

REFERENCE

[S1] Bernier, Etienne, Chantal Lavigne, and Pierre Yves Robidoux. "Life cycle assessment of kraft lignin for polymer applications." The International Journal of Life Cycle Assessment 18, no. 2 (2013): 520-528.
